# The resource requirements of perioperative patient warming in German hospitals – the results of a prospective, multicenter activity-based costing study

**DOI:** 10.1186/s12871-025-03240-6

**Published:** 2025-07-17

**Authors:** Stefan Nardi-Hiebl, Jan Wallenborn, Martin Schniertshauer, Tilo Koch, Tobias Gruebl, Alexander Torossian

**Affiliations:** 1 Department of Anesthesiology and Intensive Care Therapy, Philipps-University of Marburg, University Hospital, Marburg, Germany; 2 Department of Anesthesiology and Intensive Care Medicine, HELIOS Klinikum Aue, Aue, Germany; 3 Department of Anesthesiology and Intensive Care Medicine, University of Dresden, Dresden, Germany; 4 Gesundheitsakademie Bodensee-Oberschwaben GmbH, Weingarten, Germany; 5 Department of Anesthesiology and Intensive Care Medicine, German Armed Forces, Bundeswehr Central Hospital, Koblenz, Germany

**Keywords:** Hypothermia, Perioperative Patient Warming, Budgeting, Process Cost

## Abstract

**Background:**

Perioperative warming is essential in preventing hypothermia during surgery, a condition linked to adverse outcomes like increased infection rates, impaired coagulation, and extended hospital stays. Despite the availability of various active warming methods, such as forced-air warming (FAW) and electric warming systems, their implementation and associated costs vary significantly across hospitals, impacting resource allocation and patient care.

**Method:**

This study used a prospective, multicenter, activity-based costing approach across four German hospitals with diverse capacities. The activity-based costing model identified and measured staff time, material use, and direct costs linked to perioperative warming processes in a sample of 225 surgical patients. Warming processes were assessed across stages, including pre-warming, intraoperative warming, and post-anesthesia care.

**Results:**

Findings show significant variability in the time and cost associated with perioperative warming across institutions. The average total cost per patient between all sites ranged from EUR 3.52 to EUR 49.26, with an overall mean cost of EUR 12.29 per patient. Staff time also varied, with nurses contributing most of the required time dedicated to warming activities. At all sites, FAW was the available method during surgery, but inconsistent practices and reliance on supplemental strategies lead to considerable cost variations.

**Conclusion:**

This study illustrates potential operational and financial challenges of perioperative warming, revealing significant variability in costs and resource requirements across hospitals, influenced by institutional infrastructure, workflow efficiency and case mix. The findings also emphasize the importance of optimizing workflows and adopting best practices tailored to resource constraints. Future research should address these gaps by exploring cost-effective warming protocols, balancing efficiency with care quality, and refining workflows to enhance patient outcomes.

## Introduction

Perioperative patient warming is a crucial aspect of surgical care, primarily aimed at preventing inadvertent perioperative hypothermia—a common and significant complication in surgical patients. This condition, defined as a core body temperature below 36 °C, can lead to a range of adverse outcomes, including increased rates of surgical site infections, impaired coagulation, increased blood loss, prolonged hospital stays, and higher incidences of morbid cardiac events [10]. Anesthesiologists play a pivotal role in the implementation and management of perioperative warming strategies to mitigate these risks.

Inadvertent perioperative hypothermia occurs due to the suppression of thermoregulatory control by anesthetics, the exposure of large body surface areas during surgery, and the infusion of unwarmed intravenous fluids [3]. Various active warming systems, including forced-air warming (FAW), resistive heating, and circulating-water systems, have been developed and studied extensively to counteract these effects. Among these, FAW systems have apparently been most widely adopted and are considered highly effective [8].

The importance of active warming extends beyond temperature regulation. Active body surface warming (ABSW) systems not only maintained normothermia but also reduce shivering, and blood transfusion requirements, while improving patient satisfaction and reducing the incidence of surgical wound infections, although not affecting post-operative pain [2]. These findings underscore the multifaceted benefits of effective warming strategies in the perioperative setting.

Despite the evident benefits of perioperative warming, the prevalence of hypothermia remains high. Alfonsi et al. report that 53.5% of patients were hypothermic upon admission to the recovery room, despite the use of warming devices [1]. The study also emphasized that the combination of pre-warming and intraoperative warming was significantly more effective in preventing hypothermia than intraoperative warming alone.

In Germany, the situation mirrors the global challenge of maintaining normothermia during surgery. Gabriel et al. explored the acceptance and implementation of perioperative warming guidelines in German hospitals [7]. The study revealed that while there is a high acceptance of the German S3 Guidelines [13], which recommend strategies like pre-warming and sublingual temperature measurement, the implementation varies significantly across hospitals. Despite these variations, the study found that hospitals generally succeeded in maintaining normothermia in patients, with no postoperative hypothermia detected in a cohort of 431 patients. This highlights the importance of consistent application of warming strategies across different healthcare settings.

However, with the current budget restraints in many healthcare systems proven cost-effectiveness is a critical factor in the adoption of perioperative warming strategies. The implementation of effective warming techniques might lead to cost savings by reducing complications associated with hypothermia. Studies have shown that maintaining normothermia can decrease the incidence of surgical site infections, reduce the need for blood transfusions, and shorten hospital stays, all of which have the potential to contribute to lower healthcare costs [6, 9, 11].

While lower overall healthcare costs are beneficial for the entire system, the direct costs of patient warming play a crucial role for decision making on a hospital level as they constitute a real cash out. Warming strategies are associated with direct costs items, like equipment, consumables, maintenance and operations, implementation and finally labor. To our knowledge there has been no transparency about these cost items related to perioperative patient warming.

We hereby present the results of a prospective, multi-center activity-based costing study along the surgical patient process to collect and to analyze associated activities and cost items. The result of the study illustrates the typical resource requirements and direct costs for perioperative patient warming using various warming strategies.

## Materials and methods

The protocol was reviewed by the Institutional Review Board of the University of Marburg, Germany and approved the study as outlined (Ref. 19312). Subsequently, the study was conducted at four German hospitals by applying an activity-based evaluation approach.

### Selection of study centers

The selection of the study centers was based on their availability for such a study. We considered status of the implementation of patient warming processes according to the respective German S3 guideline. In addition, the bed capacity which broadly reflects the German hospital landscape (from a university hospital with almost 1.140 beds, a large community hospital with 1.402 beds as well as two mid-sized community hospital with 592 beds and 603 beds in private ownership (Table 1) were also taken into account. Three hospitals used pre-warmed blankets and FAW, one hospital employed an electric warming system. All hospitals use infusion warming systems (Table 2).

**Table 1:** Study sites metadata

Data item	Hospital A	Hospital B	Hospital L	Hospital M
No of beds	592	603	1,402	1,140
Type	Regular Care	Regular Care	Maximum Care	Academic
Inpatient cases per annum	24,100	13,170	44,000	43,000
Surgeries per annum	12,000	9,400	10,900	19,000
No. of operating rooms (OR)	10	6	10	30
Average OR temperature	21 deg C	21.5 deg C	21 deg C	21 deg C

**Table 2:** Available warming technologies

Area	Hospital A	Hospital B	Hospital L	Hospital M
Ward -Pre-surgery	n/a	n/a	n/a	n/a
OR—Bed holding area	n/a	PWB/PWGM	PWB	PWB
OR – Induction area	EWS	WI/partially PWB	WI/partially PWB	WI/partially PWB
OR—Surgery	FAW/WI	FAW/WI	FAW/WI	FAW/WI
PACU	FAW	PWB/FAW	PWB/FAW	PWB/FAW
Ward—Post-surgery	n/a	n/a	n/a	n/a

*PWB* Pre-warmed blanket, *PWGM* Pre-warmed gel mattress, *WI* Warm infusion

*FAW* Forced-air warming, *EWS* Electric warming system

### Study design

The study aim was restricted to resource and cost analysis; the clinical effectiveness of the various warming strategies was outside the scope of this work. The study was conducted using an activity-based costing (ABC) approach, in which relevant activities along a defined clinical process are identified, and the associated resource requirements—such as staff time and material costs—are systematically measured and recorded [4]. In the context of perioperative patient warming, ABC facilitates a structured economic analysis by allocating costs to distinct cost pools and identifying corresponding cost drivers. Typical cost pools include capital investment and maintenance for warming equipment (e.g., forced-air warming systems, fluid warmers), expenditures for single-use consumables, staff time required for setup and intraoperative management, energy consumption, and training related to protocol implementation. Key cost drivers may encompass the number and duration of procedures requiring active warming, the warming modality employed, patient-specific risk factors, adherence to institutional protocols, and perioperative staffing ratios.

To define the warming process at each hospital site, structured workshops were conducted with representatives from anesthesiology, surgery, operating room (OR) staff, and inpatient wards. These sessions served to map the perioperative warming workflow and identify cost pools, cost drivers, activities or activity phases with substantial impact on resource consumption. The findings were synthesized into a unified process model comprising six sequential phases: Ward (preoperative), OR arrival, Anesthesia, Surgery, Post-Anesthesia Care Unit (PACU), and Ward (postoperative). Specific roles and corresponding data points were assigned to each phase. Processes related to procurement, equipment maintenance, and staff training were not included in the analysis. The primary reason for this exclusion was uncertainty regarding whether a sufficiently large sample size for these activities at each hospital site could be obtained to generate meaningful data.

### Data collection

Based on the process model, an activity recording form (ARF) was developed to collect the resource requirements along the generalized process. The generalized process outlines all with patient warming associated activities a patient might encounter – from passing from the ward to the OR, receiving anesthesia, undergoing surgery, staying in the PACU and returning to the ward. For the identified activities along this process, the material used, and time stamps were recorded using the ARF. Based on these time stamps the duration of the selected activities were calculated in seconds. The ARF also distinguished between the two different professional roles, nurses and physicians, and offered the opportunity to record deviations from the standard process. The ARFs were made available in paper form to staff at the study sites responsible for patient-warming. Subsequently, the staff was also responsible for documentation. Involved staff were trained in the study concept and the ARF.

A patient was included in the study, when the patient was admitted as an inpatient, received surgery involving patient warming procedures and was under treatment of the nurses trained for the study. Only patients older than 18 years were considered. No further inclusion or exclusion criteria were defined. A pre-study sample size calculation was not conducted as meaningful input figures were not available. Therefore, we decided to collect at least 200 measurements.

Staff costs were calculated based on actual cost data from the four hospitals, averaged across all seniority levels but proportionally weighted. This yielded an average cost of EUR 0.64 per minute for nursing staff and EUR 0.84 per minute for physicians. Material costs, also averaged across the four hospitals, amounted to EUR 5.20 for one FAW blanket, EUR 1.00 for one cotton blanket, and EUR 13.40 for one HOTLINE® infusion system.

### Statistical evaluation

All data of the paper-based ARFs was recorded in a Microsoft® Access database (Microsoft® Corporation, Redmond, United States), crosschecked and transferred to the statistical software. All calculations were conducted using MedCalc® Statistical Software version 23.2.6 (MedCalc Software Ltd, Ostend, Belgium). Descriptive statistics, including means, medians, and standard deviations, were used to summarize the data. Statistical significance was determined using the Kruskal–Wallis test for all comparisons, Conover post-hoc analysis and a significance level of *p* < 0.05. Correlation factors were determined by Spearman’s ρ.

## Results

Data from 278 patient sequences were collected, whereby a (patient) sequence was defined as one passage of one patient through the entire generalized process. 53 patient sequences were excluded from data analysis because the ARFs were not entirely completed. The primary cause for non-completion of the ARFs was an emergency or other activities which required the documenting staff to refocus on other aspects. In total, 225 ARFs complied with all formal requirements and thus 225 patient sequences were analyzed. Two hospitals provided 53 completed ARFs each, one hospital 41 and the fourth hospital 78 completed ARFs.

The 225 patient sequences involved 107 male and 118 female patients with an average age of 63 years. Most patients underwent musculoskeletal surgery (88 patients, 39.1%), followed by surgery of the digestive system (35 patients, 15.6%) and surgery of the female productive system (18 patients, 8%) (Table 3).

**Table 3:** ARFs statistics

Data item	Hospital A	Hospital B	Hospital L	Hospital M
No of ARFs collected	67	88	65	58
No. of ARFs completed	53	78	53	41
Average age of patients (years)	67.0	62.3	65.1	57.0
Gender (male/female)	30 m 23 f	33 m 45 f	34 m 19 f	10 m 31 f
Type of surgeries (more than 10 surgeries)	1. Urogenital 2. Musculoskeletal 3. Vascular	1. Digestive 2. Musculoskeletal	1. Musculoskeletal 2. Male reproductive system	1. Female breast 2. Female reproductive system

**Fig. 1:**
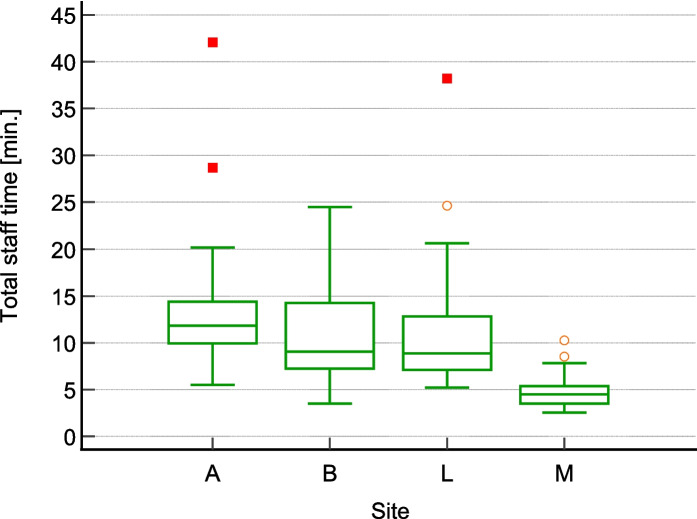
Box-whisker plot “Total staff time required”

The overall average for total staff time required for patient warming is 10.09 min (minimum = 2.53 min, maximum = 42.08 min, s = 5.52 min) (Table 4). Nurses contribute the majority of staff time dedicated to patient warming with an average of 9.91 min, whereas physicians make a much lower contribution, averaging 0.19 min. The results for total staff time across all four hospitals differ significantly. Hospitals A and M's results differ markedly from the others, while those of Hospitals B and L do not show a significant difference (Fig. 1).

**Table 4:** Results

Data item	Hospital A	Hospital B	Hospital L	Hospital M	*Overall average*
Total staff time (min.) – Average	12.92	10.45	10.91	4.71	*10.09*
Total staff time (min.)—Minimum	5.5	3.5	5.2	2.53	*-*
Total staff time (min.) – Maximum	42.08	24.5	38.13	10.25	*-*
Total staff time (min.) – StdDev	5.68	4.60	5.85	1.63	*5.53*
Nurse (min.)	12.89	10.02	10.90	4.55	*9.91*
Physician (min.)	0.28	0.43	0.01	0.16	*0.19*
Total material cost (EUR) – Average	0.90	6.24	9.43	6.59	*5.80*
Total cost (EUR) – Average	9.17	13.01	16.41	9.63	*12.29*
Total cost (EUR) – Minimum	3.52	4.59	9.53	7.82	*-*
Total cost (EUR) – Maximum	26.93	32.05	49.26	15.91	*-*
Total cost (EUR)—StdDev	3.88	5.41	8.51	1.50	*6.21*
Share of material cost in Total Cost—Average	9.8%	48.0%	57.5%	68.4%	*46.0%*
Share of Nurse cost in Total Cost – Average	90.0%	49.2%	42.5%	30.2%	*53.0%*

**Fig. 2:**
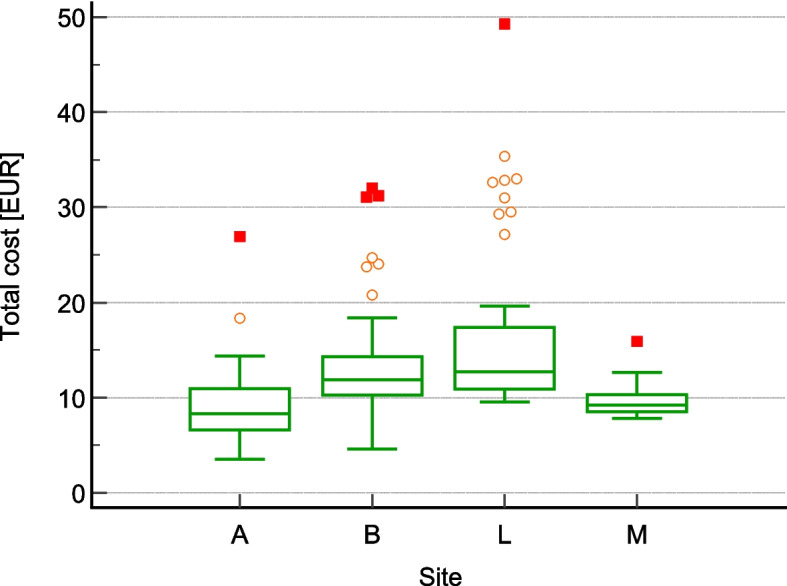
Box-whisker plot “Total cost of patient warming”

Material costs average EUR 5.80 per sequence, with total average costs at EUR 12.29 per sequence. The minimum and maximum total costs are EUR 3.52 and EUR 49.26 (s = 6.21) (Table 4). Comparing the total cost, the results of Hospital B and L differ significantly from the others, while the results of Hospitals A and M show no significant difference (Fig. 2).

During the 225 sequences recorded, 158 FAW blankets, 309 cotton blankets and 13 infusion warming systems were used. Each patient received at least one pre-warmed cotton blanket; at hospital A each patient was warmed with the electric warming system. All patients in hospitals L and M also were treated by FAW (Table 5). On average, the time interval between intubation and extubation was 148 min, the average time spent in PACU was 123 min (Table 6). Regarding the intubation/extubation time interval, hospitals A and L each differ significantly from all the other hospitals, while hospitals B and M don’t differ significantly from each other. The PACU time of hospital A differs significantly from hospital M, hospital B differs significantly from hospital L and M and hospital L just from hospital B.

**Table 5:** Material statistics

Data item	Hospital A	Hospital B	Hospital L	Hospital M
No. of patients (Sample)	53	78	53	41
No. of patients receiving PWB	n/a	78 (194 blankets)	53 (57 blankets)	41 (58 blankets)
No. of patients receiving EWS	53	n/a	n/a	n/a
No. of patients receiving FAW	7	46 (55 covers)	53 (57 covers)	41 (46 covers)
No. of patients receiving WI	0	4	9	0

**Table 6:** Time statistics

Data item	Hospital A	Hospital B	Hospital L	Hospital M	*Overall average*
No. of patients (Sample)	53	78	53	41	
*Time interval between intubation and extubation (min.)*					
Average	118	144	197	133	***148***
Minimum	23	50	76	48	***-***
Maximum	460	420	592	280	***-***
StdDev	82.78	72.21	86.20	51.79	***73.25***
*Time spent in PACU (min.)*					
Average	109	89	122	172	***123***
Minimum	0	0	0	8	***-***
Maximum	288	280	255	501	***-***
StdDev	49.16	68.27	53.10	115.01	***71.38***

**Fig. 3:**
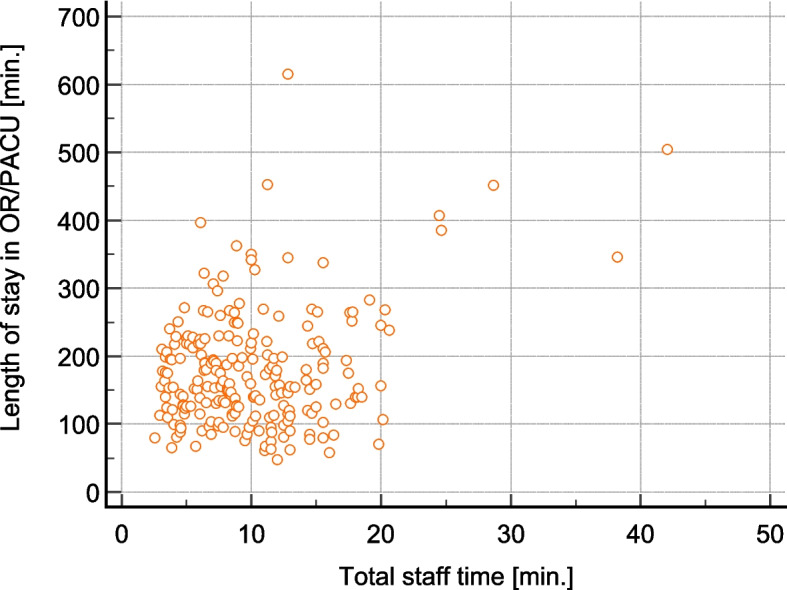
Correlation plot “Stay at OR/PACU area vs. total staff time”

**Fig. 4:**
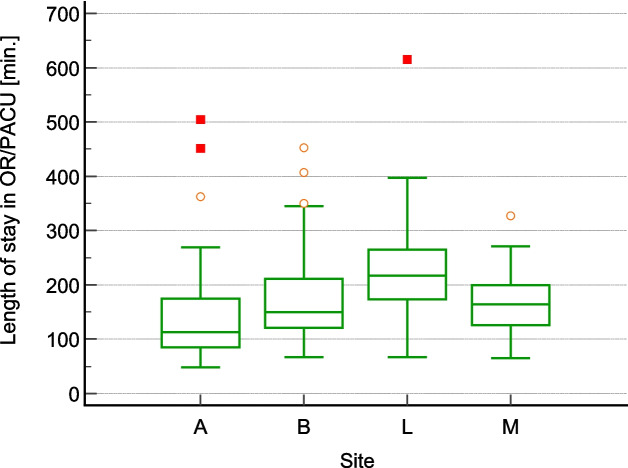
Box-whisker plot “Stay at OR/PACU area per site”

There is no observed correlation between the duration of a patient’s stay in the OR/PACU area and the total staff time required for patient warming (Fig. 3). However, the time from admission to discharge in the OR/PACU area differs significantly among the hospitals (Fig. 4).

## Discussion

This study presents the direct cost of implementing perioperative warming strategies in diverse hospital settings. Our findings underscore the resource variability required across institutions, with marked differences in material costs and staff time, particularly among hospitals with varying care capacities and resources. The wide range in total costs per patient—from EUR 3.52 to EUR 49.26—illustrates the impact of individualized institutional practices or a specific situation on expenditure, a factor that may influence decision makers’ inclination for specific warming technologies. Our study adds a nuanced perspective by detailing the direct cost impact of these protocols, showing that although comprehensive warming is beneficial, its financial feasibility remains potentially a barrier, particularly for institutions with limited budgets in the current budget climate. Considering the direct costs is important as aggressive warming could improve operating room efficiency. However, the economic benefits are significantly influenced by factors such as staff costs and the price of forced-air warming devices [12].

lthough all hospitals can make use of forced-air warming (FAW), the variations in FAW application and reliance on supplemental warming strategies reflect a lack of standardization in perioperative warming practices. This disparity in practice may lead to uneven patient outcomes, as hospitals with higher staff costs and longer warming durations might be able to achieve greater consistency in normothermia, while others could face limitations in the level of care due to resource constraints. The variation in staff time, particularly among nurses, further underscores the critical role nursing staff play in patient warming and points to the need for streamlined workflows to reduce time burden without compromising care.

The results show significant variability in resource requirements across the four hospitals, with total staff time for perioperative warming ranging from an average of 4.71 min at Hospital M to 12.92 min at Hospital A, and total costs per sequence ranging from €3.52 to €49.26. These discrepancies likely stem from differences in institutional infrastructure, workflow efficiency, and adherence to guidelines, but a specific reason could not be identified. For instance, hospitals with larger capacities and more advanced facilities, such as those equipped with pre-warming protocols and continuous temperature monitoring, tend to incur higher initial costs but may achieve better long-term patient outcomes. Conversely, smaller hospitals with fewer resources may rely more on manual processes or less efficient equipment, resulting in lower immediate costs but potentially higher rates of hypothermia-related complications. However, it must be noted that the study did not record patient-related co-morbidities or complications. Therefore, a suggested relationship between hospital size, resource level, and complication rates is hypothetical and cannot be reliably established based on the available data.

Our data confirm that material expenditure for peri-operative warming is lowest in Hospital A, which relies almost exclusively on electric warming sheets (EWS). However, Hospital A also performs a disproportionately high share of brief procedures, for which many centers forego FAW altogether. Consequently, the apparently favorable cost profile may be a function of case mix rather than inherent efficiency of EWS.

The hospitals reported partially high values for the maximum total staff time. In the case of Hospital A, this reached 42 min, which appears unusual for patient-warming activities alone. These high values were primarily caused by technical issues with the warming systems, where staff either attempted (and succeeded) to fix a fault themselves or arranged for a replacement system. Consequently, we have also recorded these rare but certainly possible occurrences. Whether the elevated staff time in Hospital A’s case is related to the use of EWS can’t be determined from the data. Also, the fact, that hospitals A and L show the highest values for staff time but also for the time interval between intubation and extubation could explain the observation.

But also variations in nursing staff availability and workload play a critical role, as shown by the high proportion of time nurses contribute to warming activities. Moreover, differences in training and familiarity with warming protocols can further amplify disparities. These findings can also serve as a benchmark to evaluate internal processes and encourage the adoption of"best-practice learning"—the transfer of evidence-based approaches tailored to specific resource constraints and capacities.

One noteworthy finding is the absence of a direct correlation between the total duration of warming and the length of stay in the OR or PACU, suggesting that while warming is essential, extending the time invested in warming activities may not significantly affect the immediate perioperative timeline. As such, we think, that the insight calls attention to the need for balancing warming efficiency with workflow demands, especially in high-turnover environments. Further research could explore optimal warming times and methods that maintain efficacy without unnecessarily increasing the patient’s OR time. It should be noted that we have not recorded whether a patient was kept longer or shorter in the PACU, for example due to bed shortages on the ward or PACU capacity constraints. However, a retrospective cursory assessment of the individual times spent in the PACU did not reveal any indications that patients included in the study were affected by such circumstances.

The environmental implications of warming practices merit attention. The widespread use of disposable materials, such as FAW blankets, contributes to medical waste, raising concerns about sustainability. In light of global efforts to reduce the environmental footprint of healthcare, exploring sustainable alternatives is crucial. Studies have shown that reusable devices, potentially also warming tools, while associated with higher initial costs, can reduce waste and long-term expenses [5]. Policymakers and hospital administrators might want to consider research and investment in environmentally friendly solutions that balance efficacy, cost, and sustainability.

This study reinforces the importance of perioperative warming process efficiency while highlighting the challenges associated with its widespread, consistent application. By providing a detailed cost analysis, we offer insights that can inform departmental heads and hospital administrators in the development of financially sustainable warming protocols that do not compromise patient outcomes. Further research focusing on cost-optimization strategies, combined with clinical efficacy studies, is essential to achieving the dual goals of resource efficiency and improved patient care in perioperative settings.

While the activity-based costing model employed in this study offers detailed insights into resource use, it has inherent limitations. For instance, it does not account for indirect costs such as staff training, equipment maintenance, or environmental impacts. Furthermore, reliance on self-reported data introduces the potential for bias, particularly in high-stress environments where documentation may be deprioritized. Regional and institutional variations, such as differences in staffing models and infrastructure, also limit the generalizability of our findings. Future studies should consider these factors to provide a more comprehensive understanding of the financial and operational aspects of perioperative warming.

It is important to note that we did not capture any patient-level temperature outcome, precluding a full cost-effectiveness analysis. Second, marked heterogeneity in case length—particularly the cluster of short operations in Hospital A—introduces confounding, because staff time and warming material use do not scale linearly with duration. Third, institutional policies (e.g. “no FAW for cases < 1 h”) were not formally documented and could not be adjusted for. These factors limit the generalizability of the absolute cost figures.

In addition, this study was conducted within the context of the German hospital system. Given the potential variability in costs and clinical workflows across different countries, a European multi-center study would be valuable to enable international comparisons of patient warming practices. Such an approach would not only facilitate the identification of context-specific strategies but also support the development of evidence-based best practices that could be adapted and implemented within individual hospital environments.

## Conclusion

The study illustrates the significant variability in workload and direct costs of patient warming across various hospital settings. The results can serve as input for the overall health-economic equation when assessing the cost-effectiveness of patient-warming strategies.

## Data Availability

The data used to support the findings of this study are available from the corresponding author upon request and approval by the rights owner.
